# Observation of the molecular genetics among children with acute lymphoblastic leukemia: A retrospective study based on the SEER database

**DOI:** 10.1097/MD.0000000000020009

**Published:** 2020-05-22

**Authors:** Ying Sun, Sili Long, Wenjun Liu

**Affiliations:** Department of Pediatrics, Laboratory of Hematologic Tumors and Birth Defects in Children, Affiliated Hospital of Southwest Medical University, Birth Defects Clinical Medical Research Center of Sichuan Province, Luzhou, Sichuan, China.

**Keywords:** acute lymphoblastic leukemia, children, molecular genetics, Surveillance, Epidemiology, and End Results database key

## Abstract

Acute lymphoblastic leukemia (ALL) is one of the most common malignancies of the hematologic system in children. Typically, ALL children with various genetic changes show different incidences, development, and prognoses. This study aimed to analyze the incidence of molecular genetic subtype among ALL children based on their clinical information, and to further investigate the relationship of genetic varieties with the prognostic factors.

From 2010 to 2016, a total of 888 ALL children with TEL-AML1 fusion gene, hyperdiploidy, hypodiloidy, IL3-IGH rearranged, E2A PBX1 fusion gene, BCR-ABL1 fusion gene, or mixed lineage leukemia (MML) rearranged were selected and analyzed through the Surveillance, Epidemiology, and End Results database.

Our results suggested that, ALL children who lived in the Northern Plains were more likely to experience genetic varieties. In addition, the TEL-AML1 fusion gene, hyperdiploidy, and hypodiloidy were more likely to be detected in ALL children aged 1 to 9 years, while MLL rearrangement was probably detected among ALL children aged <1 year. On the other hand, the 5-year overall survival varied depending on different regions (East: 42.21%; Alaska: 0.001%; Northern Plains: 1.8%; Pacific Coast: 16.3%; and Southwest: 8%), races (African American: 44.5%; white: 18.2%; and Other: 16.3%), and genetic features (TEL-AML1: 10.1%; hyperdiploidy: 19.4%; hypodiloidy: 64.7%; IL3-IGH: 0.01%; E2A PBX1: 14.2%; BCR-ABL1: 15.2%; MLL rearranged: 12.3%).

In conclusion, our study found that genetic varieties among ALL children were closely related to their prognoses, and the detection rate of genetic molecules was associated with the age, race, and living area of children.

## 1 Introduction

Acute lymphoblastic leukemia (ALL) is the most commonly seen childhood cancer worldwide, which is characterized by fever, burnout, fatigue, bone and joint pain, pale skin and mucous membrane, skin bleeding, ecchymosis, and epistaxis. A half of ALL children have liver, spleen, and lymph node enlargement. Over the past 30 years, the disease-free survival and cure rate of ALL are greatly improved thanks to the advances in diagnosis, classification, and treatment. Besides, domestic hospitals keep up with the international progresses, optimize the treatment plan according to the specific situation of the country, and achieve favorable treatment results. Optimal risk-oriented treatment can further enhance the prognosis for ALL children. In numerous developed countries, the 5-year survival rate of ALL children exceeds 90%.^[1]^ Genetic characterization, which is closely correlated with the prognosis for ALL children, plays a crucial role in the diagnosis, classification, prognosis prediction, and therapeutic decision-making of ALL.^[2,3]^. Therefore, it is of great clinical significance to further investigate the clinical characteristics of different ALL genetic phenotypes in children and to examine the influence factors of ALL as well as the prognosis for children.

The Surveillance, Epidemiology, and End Results (SEER) database of the National Cancer Institute is a national collaboration program of the United States, which involves the cancer incidence and survival data of almost 26% of all populations. Based on the SEER database, this study analyzed the incidence of molecular genetic subtype among ALL children based on their clinical information, so as to further investigate the relationship of genetic varieties with clinical characteristics, and to explore the prognostic influence factors for ALL children.

## 2 Materials and methods

### 2.1 Data collection

The patient consent and ethical approval for the study were not applicable since all the data of this study was from the SEER database, which is a public research resource.

Our study retrospectively analyzed the clinical data from 888 children diagnosed with ALL and showed 7 different genetic mutations (according to the International Classification of Diseases for Oncology), including TEL-AML1 fusion gene (9814/3ALL with w/t (12;21)(q13;q22)), hyperdiploid (9815/3), hypodiploid (9816/3), IL3-IGH rearranged (9817/3ALL with w/t(5;14)(q31;q32)), E2A PBX1 fusion gene (9818/3ALL with w/t (1;19)(q23;q13.3)), BCR-ABL1 fusion gene (9806/3 ALL with w/t (9;22)(q34;q11.2), and MLL rearranged (9807/3ALL with w/t (v;11q23)) derived from the SEER database (https://seer.cancer.gov/). Typically, the information of gender, time of diagnosis, age at diagnosis, race, and region was collected. According to the SEER database, race was classified as white, African American, or other (such as American Indian/Alaska Native and Asian/Pacific Islander); while region was categorized as Alaska, Pacific Coast, East, Southwest, and Northern Plains. Besides, the following patient data were also collected:

1. the age at diagnosis of 0 to 19 year-olds;
2. the time of diagnosis in the year 2010 to 2016;
3. initial treatment of chemotherapy (annotated as yes); and
4. children with positive histological diagnosis.

The case exclusion criteria were as follows:

1. children with incomplete follow-up information;
2. those with incomplete survival time;
3. those who died within 30 days; and
4. those with the follow-up length of < 30 days.

### 2.2 Statistical analyses

In this study, the Chi-squared test was used to compare the clinical characteristics of all enrolled patients, and to determine the differences between different metastatic lesions. In addition, the SEER Stat software was utilized to calculate the age-adjusted incidence rate per 100,000 population, as well as the incidence rate ratio (IRR) following the US population in 2000. Notably, the age-adjusted rate is a weighted average of the crude rates, in which the weights represent the proportions of persons in corresponding age groups of a standard population. IRR was obtained through dividing the rate of a group by that of the reference (first) group, and the ratio *P*-value together with the relevant 95% confidence interval was utilized to describe the subgroup significance. The overall survival (OS) was estimated according to the Kaplan–Meier method, the differences between different groups were compared by the log-rank test, and the survival time as well as status was derived from the SEER 18 Regs Custom Data. Specific factors that affected ALL were identified through multivariate Cox regression analysis. *P*<.05 (2-sided) was deemed as statistically significant.

## 3 Results

### 3.1 Patient characteristics

Altogether 888 children diagnosed with ALL were identified from 2010 to 2016, and these patients were associated with positive TEL-AML1 fusion gene, hyperdiploidy, hypodiploidy, IL3-IGH rearranged, positive E2A PBX1 fusion gene, positive BCR-ABL1 fusion gene, or MLL rearranged. The median age of the patient cohort was 8.5 years (range, 0–19 years). Table 1 displays the demographic and clinical characteristics of the included patients.

**Table 1:**
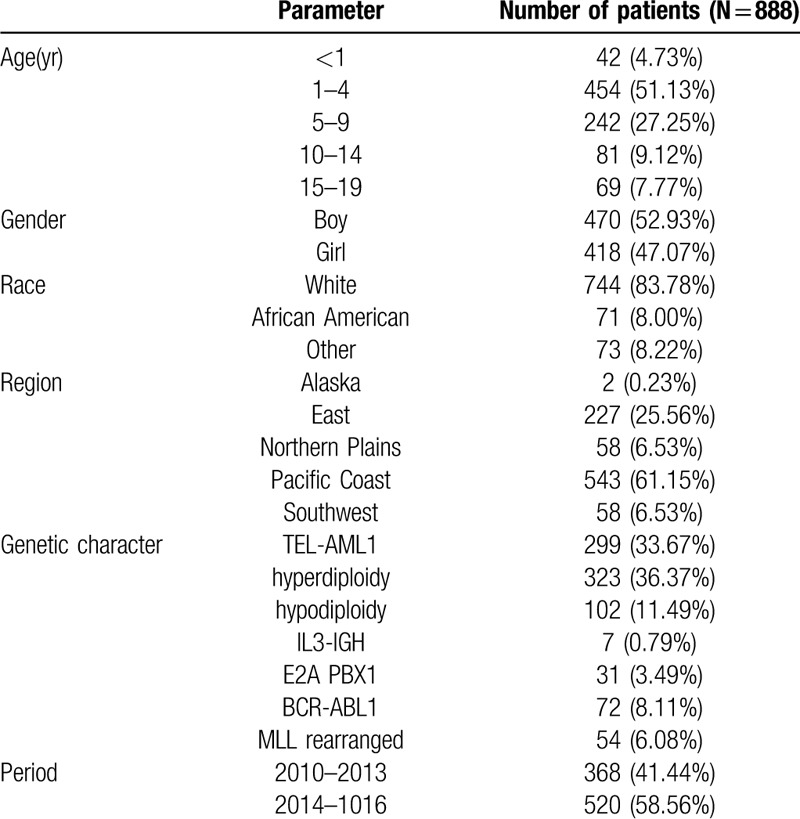
Demographical characteristics of the included patients.

### 3.2 Incidence of ALL

Table 2 displays the variations in different genetic ALL incidences based on demographic characteristics. Clearly, the proportion of ALL children aged <10 years with genetic changes was remarkably higher than that of those aged > 10 years. Typically, children aged 1 to 4 years showed the highest incidence (IRR range, 1.981–3.827, *P*< .001). Differences in the incidence rates obtained based on gender and residential region were not statistically significant. Moreover, the incidence markedly increased in whites, and the genetic changes in ALL children were shown as follows. As observed, more ALL children were associated with TEL-AML1 fusion positive and hyperdiploidy.

**Table 2:**
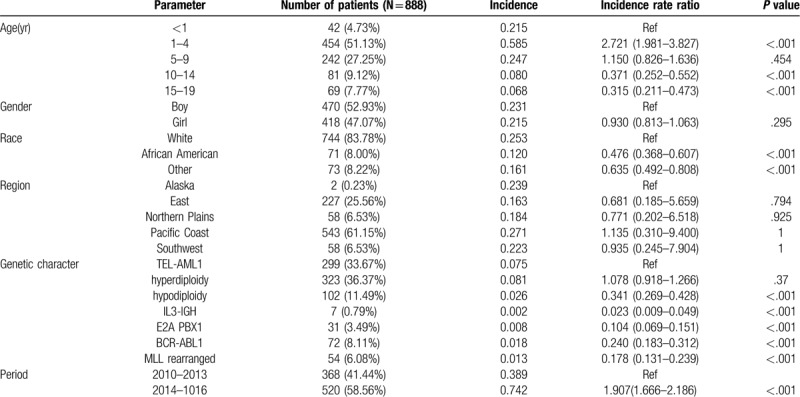
The variations in different genetic ALL incidences based on demographical characteristics.

### 3.3 Clinical characteristics

**Figure 1:**
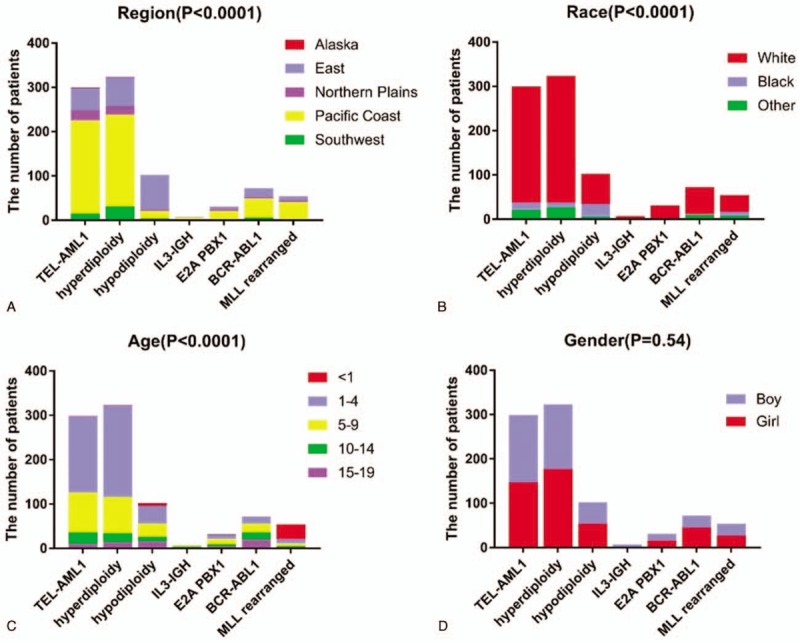
. Genetic changes and clinical characteristics. A. Detection rate of abnormal gene in acute lymphoblastic leukemia (ALL) children among different regions; B. Detection rate of abnormal gene in ALL children among different races (other included American Indian/Alaska Native, and Asian/Pacific Islander); C. Detection rate of abnormal gene in ALL children among different ages; D. Detection rate of abnormal gene in ALL children among different genders.

Generally, genetic molecular changes are closely related to the clinical characteristics of children. In our study, ALL children who lived in Alaska and Northern Plains were more likely to experience genetic changes (Fig. 1A). In addition, white ALL children also suffered from higher rates of genetic changes (Fig. 1B). Besides, TEL-AML1 fusion gene positive, hyperdiploidy, and hyperdiploidy were more likely to be detected among ALL children aged 1 to 9, while those aged <1 year were associated with a higher rate of while MLL rearrangement (Fig. 1C). However, there was no significant statistical significance in gender among various genetic change types (Fig. 1D).

### 3.4 Survival time of ALL children

**Figure 2:**
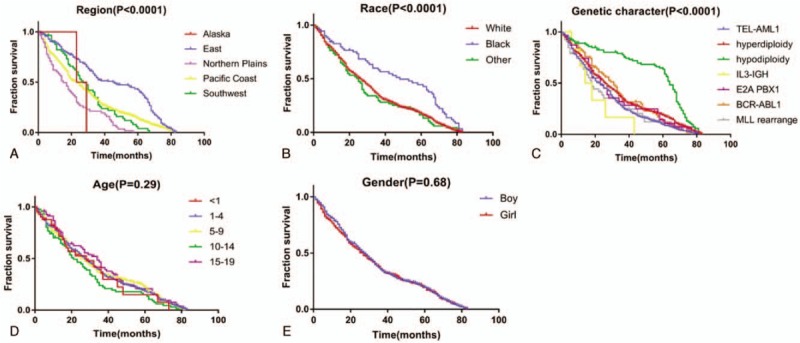
. Survival time of acute lymphoblastic leukemia (ALL) children with different clinical characteristics. A. Survival time of ALL children among different regions; B. Survival time of ALL children among different races; C. Survival time of ALL children among different genetic characteristics; D. Survival time of ALL children among different ages; E. Survival time of ALL children among different genders.

According to the clinical data, the median survival of this patient cohort was 50 months. The living region, race and genetic changes among ALL children were closely correlated with their survival time. In addition, the prognosis for children from the East was favorable relative to that for children from other regions (5-year survival rate: Alaska–0.001%; Northern Plains–1.8%; Pacific Coast–16.3%; and Southwest–8%), with a median survival of 45 months and a 5-year survival rate of up to 42.21% (Fig. 2A). The survival time of African American children was also remarkably higher than those of the white and other racial groups (5-year survival rate: African American–44.5%; white–18.2%; other–16.3%) (Fig. 2B). Additionally, gene mutation was also an important factor affecting patient prognosis. Children with hypodiploid ALL had the best prognosis, while those with IL3-IGH rearranged had the worst prognosis (5-year survival rate: TEL-AML1, 10.1%; hyperdiploidy, 19.4%; hypodiloidy, 64.7%; IL3-IGH, 0.01%; E2A PBX1, 14.2%; BCR-ABL1, 15.2%; MLL rearranged, 12.3%) (Fig. 2C). Nonetheless, the prognosis showed marked difference among various age and gender groups (Fig. 2D-E).

### 3.5 Multivariate analysis of OS

Table 3 shows the multivariate analysis for all patients. Typically, the living regions and the time of ALL incidence were identified as the independent predictors of OS. Besides, the results of multivariate analysis revealed no significant difference in either genetic characteristics or OS based on age, gender, and race.

**Table 3:**
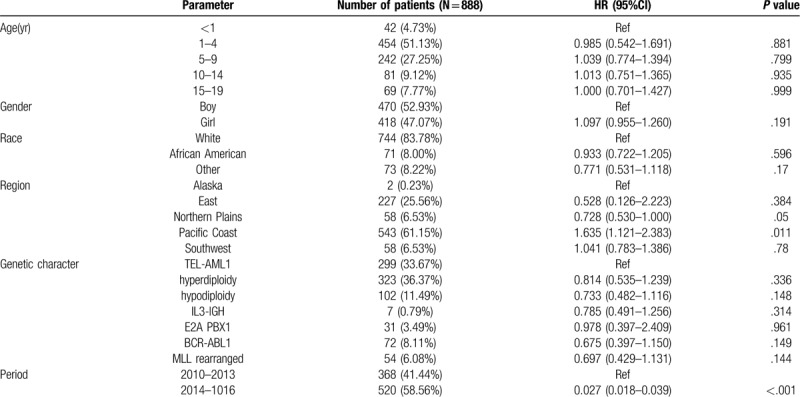
Multivariate analysis of overall survival of included patients.

## 4 Discussion

At present, more intensive studies have been carried out on ALL children, and the abnormal karyotype and the expression of related fusion genes have become the important independent prognostic factors. In addition, their different molecular biology and genetic characteristics also become the markers in recurrence monitoring, prognosis evaluation and changes in disease observation.^[4–6]^ In 2010 to 2016, a total of 888 ALL children with genetic changes were diagnosed in the United States (Table 1), with the overall incidence of ALL of 0.223 cases per 1 million.

The TEL-AML1 fusion protein, which is also known as ETV6-RUNX1, is induced by t (12;21) (p13; q22) translocation, and it is detected in 25% children patients with B-cell-precursor acute lymphoblastic leukemia (BCP-ALL). In China, the positive rate of TEL/AML1 fusion gene is about 19.8%, which is the most common fusion protein in childhood cancer.^[7,8]^ In addition, the incidence rate of TEL-AML1 fusion gene is 0.075 per million, and it is the most extensively detected gene variation in this study. TEL-AML1 fusion gene is also expressed in healthy newborns too,^[9]^ and its pathogenesis is mainly associated with a “two-hit” model.^[10]^ DNA repair takes place following TEL and AMLl disruption when the TEL gene HLH region and the almost entire AMLl gene are spliced together. Later, the TEL-AMLl fusion gene is formed, which functions to inhibit the transcriptional activity, thus affecting the self-renewal and differentiation of hemopoietic stem cells. Many clinical studies have shown that ALL children with TEL-AML1 fusion gene have superior prognosis.^[8]^ Nonetheless, the prognosis for ALL children with TEL-AMLl fusion gene-positive in this study is relatively poor. This is because that only children with genetic variations are enrolled in this study, while those without gene mutation are not enrolled for comparison (Fig. 2C). Therefore, the effect of TEL-AMLl fusion gene on the prognosis for ALL children should be further explored combined with specific chemotherapy regimens, related clinical test indexes, recurrence, and other complications.

Additionally, the incidence of changes in chromosome number among ALL patients is also relatively high. Typically, the hyperdiploid karyotype in ALL refers to the chromosome number in leukemia cells of > 50 and usually < 66, which is common among children and accounts for 25% B-ALL cases. Nonetheless, it is rare in infants and young children, and the incidence decreases with age, which is consistent with our results (Fig. 1C). The increase in number is the most common chromosome abnormality, which follows the order of chromosomes 21 > X > 14 > 4 > 1 > 2 > 3. Children with hyperdiploid karyotype ALL generally have favorable prognosis, and the cure rate is over 90%; particularly, chromosomes 4, 10, and 17 are trisomy at the same time.^[11]^ The karyotype of ALL with subdiploid refers to the chromosome number of < 46 in leukemia cells, of which the chromosome number of 24 to 31 is near haploid, that of 32 to 39 is low subdiploid, and that of 40 to 45 is near diploid. Subdiploid ALL accounts for 5% of all ALL cases, while that with the chromosome number of < 45 occupies about 1% of all ALL cases, and it can be seen in both children and adults, but near haploid (23–29 chromosomes) is mainly found in children. The clinical manifestations, morphology and cytochemical characteristics are similar to those of other ALL types, and CD19 and CD10 are often positive. It is found that subdiploid B-ALL is associated with poor prognosis, but the prognosis for the chromosome number of 44 or 45 is the optimal, while that for near haploid is the poorest.^[12,13]^ However, our results showed that the prognosis for subdiploid ALL children was markedly superior to that for hyperdiploid ALL cases (Fig. 2C), which might be because that, the sample size for studying the changes in subdiploid was evidently lower than that for hyperdiploid, and that the near haploid or small number of subdiploids was easily missed in routine karyotype analysis.^[11]^ Therefore, the chromosome karyotype analysis method should be further improved, and the relationship of chromosome karyotype changes with the prognosis for ALL should be accurately analyzed according to the number of specific chromosome changes.

The incidence of BCR-ABL1 is low in children, which accounts for 2% to 4% of all ALL cases; besides, the morbidity increases with age, and takes up 25% of adult ALL cases. Such type of ALL has the worst prognosis among all age groups, and its form is the same as other types of ALL. Typically, such patients express CD10, CD19 and TdT; often, myeloid antigens CD13 and CD33 are expressed simultaneously.^[14]^ To the best of our knowledge, there are relatively few ALL children with BCR-ABL1 fusion gene, and only 72 cases were included in this study. According to our results, such patients had relatively poor prognosis, which was consistent with the above conclusions (Fig. 2C).

The MLL gene is located on chromosome 11q23 and consists of 36 exons. In addition, it is homologous to the thithorax gene of Drosophila melanogaster, and encodes a protein possessing 3969 amino acids, with a relative molecular weight of 430∗103. At present, over 60 types of MLL gene translocation rearrangements have been identified, and about 30 partner genes have been identified. Translocation often leads to fusion of MLL gene with various partner genes. Specifically, MLL/AF4, MLL/ENL, MLL/AF9, MLL/ELL, MLL/AF6, and MLL/AF10 are the common fusion genes.^[15]^ It has been confirmed in some studies that, leukemia with MLL rearrangement is characterized by the clinical, hematological, and prognostic features, such as the high peripheral white blood cell count, common organ infiltration, frequent central nervous system leukemia, mild mitigation through routine chemotherapy, high recurrence rate after remission, poor prognosis, short average survival time.^[16]^ In this study, only 57 ALL children with MLL rearrangement had relatively poor prognosis. Further typing of fusion genes and biochemical results was not carried out in this study due to the insufficient clinical data from the database; nonetheless, our results suggested that the proportion of mutations in this gene remarkably increased in children aged less than 1 year (Fig. 1C).

ALL with E2A-PBX1 fusion gene accounts for 6% of all children ALL cases, and it is also detected in adults, but the incidence is lower than that in children. The typical phenotypes are CD19 and CD10 expressed by Pre-B-ALL, but not all cases belong to the Pre-B-ALL phenotypes. It has been reported that, the E2A-PBX1 fusion gene in 25% cases is produced by t (1;19) (q23;p13.3), and patients with E2A-PBX1 fusion gene experience a higher incidence of CNS relapse.^[17]^ Notably, the occurrence of such disease is related to the function of E2A-PBX1 fusion gene in inhibiting the normal transcription factors E2A and PBX1. In our study, the incidence of E2A-PBX1 fusion gene was 0.008 per 1 million, which was associated with the relatively dismal prognosis compared with that in other children with variant ALL.

BCP-ALL with eosinophilia and IL3-IGH rearranged (t(5;14)(q31;q32)) is a rare disease, which accounts for <1% of all ALL cases.^[18,19]^ In this study, only 6 cases were detected with BCP-ALL. To the best of our knowledge, few BCP-ALL cases are reported, which has hindered the comprehensive evaluation on its prognosis, especially for the advances in the therapeutic management of BCP-ALL. Derrieuxc reported a case with poor response to chemotherapy, chemoresistance, induction failure, high MRD levels and cytological relapse.^[20]^ Our study suggested that, the prognosis for ALL children with IL3-IGH rearranged was poor.

According to our results, the detection rate of ALL among children with genetic abnormalities was correlated with their age, race, and genetic characteristics. Besides, ALL children living in Pacific Coast were linked with a relatively large number of genetic changes in this study, which, might be because that, patients from Pacific Coast area were injected into the database. The detection rate of gene abnormalities among ALL children aged 1 to 9 years dramatically increased. Additionally, studies in several regions (including North India, China, and the United States) indicate that the high incidence of ALL in children aged 1 to 9 years may be related to our results.^[21–24]^ We speculated that the mechanism of ALL gene variation might be related to the growth and development of children, living environment, physiological, and psychological changes, as well as other factors. In addition, our results indicated that the detection rate of ALL in children aged 1 to 9 years was quite high, which was consistent with conclusions from Shen and Kahn.^[22,23]^ Moreover, the detection rate was the highest among ALL children aged 1 to 4 years, and that of ALL children with hyperdiploidy also dramatically increased, further suggesting that hyperdiploidy was the most common cytogenetic subtype of childhood ALL.^[25–27]^. Gender was also implicated in the prevalence of ALL,^[21–24]^ nonetheless, our study discovered that the detection rate of gene abnormalities showed no marked correlation with gender. The incidence of ALL evidently increased in whites, which was consistent with findings from Kahn. The unequal access to these advances might potentially give rise to the survival disparities between groups as well as between biologic and non-biologic factors (such as medication adherence, disease biology, and pharmacogenomics)^[28]^; alternatively, it might be ascribed to the difference in the access to healthcare. Meanwhile, it was consistent with the results from 1992 to 2013.^[29]^ In addition, our study discovered that the prognosis for the whites was worse, whereas the ETV6-RUNX1 fusion gene and hyperdiploidy were more likely to be detected in the white ALL children. Similar patterns of survival disparities are also observed using the population-based data from the California Cancer Registry (CCR), which indicates increased hazard ratios of death among African American (by 57%), Hispanic (by 38%), and Asian children (by 33%) compared with that in the white children.^[30]^

Some limitations should be noted in this study, including the retrospective study design, lack of information on the received therapy, as well as on the prognostic factors such as white blood cell count, MRD status, and comorbidities. Nonetheless, our study provided important real-world information on the incidence and outcomes of ALL children using a national population-based registry. In addition, only 7 genetic abnormal genes mentioned above were included in this study, and the clinical information for other ALL children with unclear genetics or with no genetic changes was lacking. Combined with the classification based on more clinical data, the characteristics of ALL children with various genetic abnormalities should be summarized and analyzed, so as to realize the Jin quasi-treatment and to improve the treatment plan as well as prognosis for patients.

In summary, gene abnormality is closely correlated with the occurrence, development, and prognosis for ALL children, and the clinical characteristics vary among different genetic varieties. Our study finds that the detection rates of ALL with TEL-AML1 fusion gene and hyperdiploidy are higher in children living in the Pacific Coast. On the other hand, the prognosis for children patients living in the East is superior to that for children aged <1 year, and children aged >10 years have worse prognosis. Besides, the prognosis for the African-American ALL children are relatively better. On this basis, the diagnosis and treatment schemes should be further investigated to improve the prognosis and enhance the quality of life of ALL children according to more clinical characteristics of ALL children with different genetic characteristics.

## Author contributions

**Conceptualization:** Ying Sun, Wenjun Liu.

**Data curation:** Ying Sun, Sili Long.

**Formal analysis:** Sili Long.

**Funding acquisition:** Wenjun Liu.

**Investigation:** Ying Sun.

**Methodology:** Sili Long, Wenjun Liu.

**Resources:** Ying Sun, Wenjun Liu.

**Software:** Sili Long.

**Supervision:** Wenjun Liu.

**Writing – original draft:** Ying Sun, Sili Long, Wenjun Liu.

**Writing – review & editing:** Wenjun Liu.
